# Hidradenitis suppurativa is associated with cardiometabolic comorbidities in a racially and ethnically diverse safety net population: A cross-sectional analysis

**DOI:** 10.1016/j.jdin.2024.10.003

**Published:** 2024-10-28

**Authors:** Mariano Alba, Nora Rudd, Adam Zakaria, Aileen Y. Chang, Erin H. Amerson

**Affiliations:** a University of Nevada, Reno School of Medicine, Reno, Nevada; b School of Medicine, University of California, San Francisco, California; c Department of Dermatology, University of California, San Francisco, California; d Department of Dermatology, Zuckerberg San Francisco General Hospital and Trauma Center, San Francisco, California

**Keywords:** cardiometabolic comorbidities, diabetes, hidradenitis suppurativa, hypertension, hyperlipidemia, obesity, racial/ethnic minorities

*To the Editor:* Hidradenitis suppurativa (HS) is a systemic inflammatory disease that disproportionately impacts patients of color in the United States.^1^ Previous studies identified associations between HS and the cardiometabolic comorbidities (CMCs) diabetes, hypertension, and hyperlipidemia,^2^ but most analyzed predominantly White populations,^1^ and only 2 controlled for obesity.^3^^,^^4^ Given the complex and poorly understood relationships between HS, obesity, race/ethnicity, socioeconomics, and CMCs,^1^ we analyzed CMC rates in patients with and without HS among a racially/ethnically diverse safety net population after controlling for obesity.

We performed a cross-sectional study of patients at Zuckerberg San Francisco General Hospital from August 2019 to June 2023 (IRB#20-33109). We compared rates of CMCs (diabetes, hyperlipidemia, hypertension, and obesity [Body mass index > 30]), between patients with and without HS matched 1:2 on patient-reported gender, age, and race/ethnicity. Patients with HS were identified by International Classification of Diseases (ICD)-10 code and verified through chart review. Continuous variables were compared using simple Student *t* test, categorical variables were compared using χ^2^ testing. We investigated the association of HS (exposure) with diabetes, hypertension, and hyperlipidemia (outcomes) after controlling for patient-reported gender, age, race/ethnicity, obesity, and smoking status (covariates) in multivariable logistic regression. A *P*-value < .05 was significant, and analysis was performed in STATA.

Our analysis included 303 patients with HS and 606 patients without HS (38% Black, 34% Hispanic, and 87% publicly insured). Patients with HS had higher rates of obesity (74% vs 50%, *P* < .01), diabetes (39% vs 19%, *P* < .01), and hypertension (46% vs 32%, *P* < .01), but not hyperlipidemia (39% vs 34%, *P* = .11) (Table I). After controlling for covariates, HS patients had 2.32 times the odds of diabetes (*P* < .01, 95% CI [1.63-3.30]) and 1.66 times the odds of hypertension (*P* = .01, 95% CI [1.15-2.41]) compared with patients without HS (Table II).

**Table I:** Demographics and laboratory characteristics of study population

Characteristics	Patients with HS∗, n (%)	Patients without HS, n (%)	*P*
No.	303	606	
Age (y), mean ± SD	43 ± 14	44 ± 19	.52
Patient-reported gender			.88
Men	99 (33)	201 (303)	
Women	204 (67)	405 (67)	
Race/ethnicity			.82
Non-Hispanic Asian	20 (7)	50 (8)	
Non-Hispanic Black	116 (38)	233 (38)	
Non-Hispanic White	44 (14)	81 (13)	
Hispanic	103 (34)	209 (34)	
Non-Hispanic Other†	15 (5)	28 (3)	
Unknown	5 (2)	5 (1)	
Insurance status			.15
Healthy San Francisco‡	26 (8)	76 (12)	
Medicare	51 (17)	68 (11)	
Medi-cal	206 (68)	421 (69)	
Commercial	4 (1)	7 (1)	
None	16 (5)	27 (4)	
Smoking status			<.01
Never	121 (40)	353 (58)	
Current	103 (34)	112 (18)	
Former	73 (24)	111 (18)	
Unknown	6 (2)	27 (4)	
	Mean (95% CI)	Mean (95% CI)	

BMI§ kg/m	36.7 (35.5-37.8)	31.7 (31.0-32.4)	<.01
Hemoglobin A1c %	6.8 (6.6-7.1)	6.1 (6.0-6.2)	<.01
Highest total cholesterol mg/dL	191 (185-196)	195 (191-198)	.21
Lowest high density lipoprotein mg/dL	49 (47-50)	54 (53-55)	<.01
Highest low density lipoprotein mg/dL	110 (106-114)	113 (110-116)	.28

*BMI*, Body mass index; *HS*, hidradenitis suppurativa.

∗ Included on the problem list or discussed in a dermatology or primary care note.

† Other racial group defined as self-identifies as American Indian, Alaska Native, Native Hawaiian, other Pacific Islanders, or 2 or more races.

‡ Healthy San Francisco enables uninsured residents of San Francisco who are not Medicare or Medicaid eligible to access medical services in San Francisco County.

§ BMI was determined based on the most recent recorded height and weight and calculated as weight in kilograms divided by height in meters squared.

**Table II:** Comparison of cardiometabolic comorbidities after controlling for obesity and smoking status

Characteristics	Patients with HS, n (%)	Patients without HS, n (%)	OR∗ (95% CI)	*P*
Overall (*n =* 909)				
Hypertension†	139 (46)	194 (32)	1.66 (1.15-2.41)	.01
Hyperlipidemia‡	119 (39)	205 (34)	1.22 (0.87-1.71)	.24
Diabetes§	118 (39)	117 (19)	2.32 (1.63-3.30)	<.01

*HS*, Hidradenitis suppurativa.

∗ We compared differences between patients with HS and patients without HS after adjustment for patient-reported gender, age, obesity, and smoking status. We also adjusted for race/ethnicity in the comparison involving the overall study sample.

† Two or more blood pressure readings >130/80 mm Hg in outpatient visit (not including emergency department, urgent care, procedure visit, or hospital).

‡ Based on presence of ICD-10 code and confirmed via presence on problem list or in assessment and plan section of provider note.

§ Based on presence of ICD-10 code and confirmed via presence on problem list or in assessment and plan section of provider note, excluding gestational diabetes.

We found patients with HS had higher odds of diabetes and hypertension when compared with those without HS among a diverse safety net population after adjusting for obesity and smoking. Associations with diabetes and hypertension were stronger compared with previous studies that also accounted for obesity, whereas the association with hyperlipidemia did not reach statistical significance.^3^^,^^4^ Disparities may be attributed to methodologic differences in CMC definitions, together with differences among the study populations regarding baseline comorbidity status and HS severity. Current United States Preventive Services Task Force guidelines recommend only screening for diabetes among adult patients who are overweight or obese.^5^ Based on our findings, it may be reasonable for patients with HS to undergo diabetes screening and blood pressure monitoring regardless of weight status.

Strengths of this study include a diverse safety net population and comorbidities confirmed by chart review rather than ICD code alone. Our cross-sectional approach could not determine causality. Only 303/604 (50.2%) patients with HS identified during our study period had data on all 4 comorbidity measurements, likely representing more engaged patients. Generalizability may be limited by small sample size from a single institution. Race/ethnicity are socially constructed, and differences in HS and its comorbidities stem primarily from socioeconomic rather than genetic factors; thus, we did not stratify results by race/ethnicity. Future multicenter studies examining diverse cohorts should further explore these associations.

## Conflicts of interest

None declared.
